# Off-label use of targeted therapies in osteosarcomas: data from the French registry OUTC’S (Observatoire de l’Utilisation des Thérapies Ciblées dans les Sarcomes)

**DOI:** 10.1186/s12885-015-1894-5

**Published:** 2015-11-05

**Authors:** Mathilde Penel-Page, Isabelle Ray-Coquard, Julie Larcade, Magali Girodet, Laure Bouclier, Muriel Rogasik, Nadège Corradini, Natacha Entz-Werle, Laurence Brugieres, Julien Domont, Cyril Lervat, Sophie Piperno-Neumann, Helène Pacquement, Jacques-Olivier Bay, Jean-Claude Gentet, Antoine Thyss, Loic Chaigneau, Bérangère Narciso, Helène Cornille, Jean-Yves Blay, Perrine Marec-Bérard

**Affiliations:** Department of Pediatric Oncology, Léon Bérard Cancer Center, 28, rue Laennec, 69008 Lyon, France; Université Claude Bernard Lyon 1, Lyon, France; Hôpital Mère-enfant, 7 quai Moncousu, Nantes, France; CHU Hautepierre, 1 avenue Molière, Strasbourg, France; Institut Gustave Roussy, 114 Rue Edouard Vaillant, Villejuif, France; Centre Oscar Lambret, 3 rue Frédéric Combemale, Lille, France; Institut Curie, 26 rue d’Ulm, Paris, France; Centre Jean Perrin, 58 Rue Montalembert, Clermont-Ferrand, France; CHU La Timone, 264 rue Saint-Pierre, Marseille, France; Centre Antoine Lacassagne, 33 Avenue Valombrose, Nice, France; CHU Jean Minjoz, 2 Bd Fleming, Besançon, France; CHU Bretonneau, 2 Bd Tonnellé, Tours, France; CHU Raymond Poincaré, 104 Bd Raymond Poincaré, Garches, France

**Keywords:** Targeted therapy, Tyrosine-kinase inhibitors, Off-label, mTOR inhibitors, Bone sarcoma, Osteosarcoma, Relapse, Maintenance therapy

## Abstract

**Background:**

The objective of this study is to explore the off-label use of targeted therapies (TTs) for patients with osteosarcoma registered within the French Sarcoma Group – Bone Tumor Study Group (GSF-GETO) national registry.

**Methods:**

All patients with an osteosarcoma, registered between January 1, 2009 and July 15, 2013 were analyzed.

**Results:**

Twenty-nine patients with refractory relapsed osteosarcomas received 33 treatment lines of TTs. The median age at the beginning of treatment was 19 years (range 9–72). The median number of previous lines of chemotherapy was 3 (range 1–8). Before inclusion, 3 patients were in second complete remission, 26 were in progression for metastatic relapse. Twenty-three patients received sirolimus (in combination with cyclophosphamide for 18); 5, sunitinib; 4, sorafenib; and one, pazopanib. Stable disease was observed for 45.5 % of patients (95 % Confidence Interval (CI) [20–52.8]). The median Progression-Free Survival (PFS) was 3 months (95 % CI [2–5.4]) for patients treated by sirolimus and 1.8 months (95 % CI [1.3–2.8]) for patients receiving multi-targeted tyrosine kinase inhibitors; 6-month PFS 15 %. The median Overall Survival (OS) was 6.8 months (95 % CI [4.7–12.1]), and one-year OS was 24 %. In a multivariate analysis, PFS was superior for patients receiving sirolimus compared to other TTs (Hazard Ratio (HR) = 2.7, 95 % CI [1.05–7.1]). No toxic death was reported. Grade 3 and 4 toxicities were observed in 27 and 6 % of cases respectively.

**Conclusion:**

Off-label TTs, especially sirolimus, reported benefit in the treatment of refractory osteosarcomas with an acceptable toxicity profile, including in pediatric population.

## Background

High-grade osteosarcoma is the most common malignant bone tumor in adolescents and young adults [1]. Multimodal treatment including chemotherapy and radical surgery increased the Progression-Free-Survival (PFS) from 10 to 65 % [2]. However, we still observe 30 % of relapse, mainly with metastatic stage, with less than 20 % long-term survival for these patients [3].

The role of chemotherapy in recurrent osteosarcomas is not fully established [4]. There is no standard regimen recommended for second-line treatment [1, 5]. Except for muramyl tripeptide (L-MTP-PE) which demonstrated an improvement of median time to relapse from 4,5 months to 9 months in a phase II trial [6], recently tested drugs (etoposide, carboplatine, gemcitabine, high dose chemotherapy [7], ecteinascidin [8], samarium [9]) failed to improve long-term survival of these patients [10, 11].

Several biological pathways are implicated in bone sarcomas and represent a potential interesting approach for the treatment of such tumors with targeted therapies (TTs) : sustaining proliferative signal (IGFR, SHH/GLI, PDGFR, c-KIT), evading cell growth suppressors (p53, RB, CDK), resisting to cell death (ERK activation, proapoptotic molecule inhibition, antiapoptotic molecule activation Bcl2, syndecan-2), enabling replicative immortality, increasing angiogenesis (VEGFR, IGFR, PDGFR, HIF1α) and activating invasion and metastasis, genome instability (p53, GADD45), evading immune destruction (IFN), or interacting with the bone microenvironment (RANK/RANKL/OPG) [12]. Unfortunately, the rarity of these pathologies and the specificity of the pediatric population don’t hold pharma industries nor governments to delineate phase III trials and prove the benefit of such compounds for refractory osteosarcomas.

In 2008, the GSF-GETO established a National Observatory for The off-label Use Of Targeted Therapies in Sarcomas (OUTC’S) as a resource for the research into the use of TTs in routine practice. All medical data regarding the use of off-label TTs in sarcomas was collected in a prospective way to analyze activity and toxicity of TTs in these tumors [13]. This report aims to describe the utilization, efficacy, and safety data on osteosarcoma patients registered in OUTC’S in order to identify TTs which warrant further investigations in this pathology.

## Methods

### Patients/Registry

Patients who met the following criteria were included: osteosarcoma upon histological diagnosis, no age-limit, not amenable to curative treatment or inclusion in clinical trial, treatment in France. They received an information letter. Oral consent for data collection and use for research purpose was requested before inclusion. Children could be included with parents’ oral consent. As reported previously, all details of the methodology was anticipated. Once a patient registered, he was evaluated by his referring doctor and a follow-up file was sent every two months to the coordination center.

### Competent authorities approval

All data was collected by the coordination center (Centre Léon Bérard, Lyon) upon approval of the local Clinical Trial Review Committee (CREC, Lyon, France), the French Consultative Committee for the Data Processing in Health Research (CCTIRS, Paris, France) and the French data protection authority (National Commission of Informatics and Liberty, Paris, France, declaration n°1375805). Most decisions of treatment involving off-label TT treatment were made during a Multidisciplinary Tumor Board (MTB), as defined by the French Sarcoma Network (NetSarc) [14].

### Data collection and study endpoints

The primary objective was to describe the efficacy of off-label TTs in osteosarcoma patients. Endpoints were response rate for each TT: rate of complete and partial remission (CR, PR) according to RECIST (Response Evaluation Criteria for Solid Tumors [15]), disease control rate (rate of CR, PR and stable disease as best response), Progression-Free-Survival (PFS), Overall Survival (OS) and duration of response. The secondary objective was the characterization of toxicities.

### Statistical methods

PFS was calculated from the beginning of TT to the date of the event, defined as the first documented progression or death whatever the cause under treatment. Patients who did not experience an event were censored at the date of treatment discontinuation or at the date of last contact for patients still under treatment. OS was calculated from the beginning of treatment until the date of death whatever the cause, and censored at the date of last contact for patients alive. PFS and OS were estimated by the Kaplan Meier method with their 95 % confidence interval (CI) and comparisons were done by a logrank test, in the XLstat software. Safety evaluation was based on the frequency and severity of toxicities graded according to the Common Terminology Criteria for Adverse Events [16].

Patients could be included in the Observatory for each consecutive line of TT. All analyses were performed on the total number of treatment lines, except for data regarding OS which was analyzed on the total number of patients included at least once in the study. Regarding patients included several times, OS was defined as the time between the first inclusion and date of the last follow up for the last treatment. The database was locked for statistical analysis in July 2013. This is a descriptive analysis.

## Results

### Patient characteristics

From September 2009 to July 2013, 29 patients from 12 centers (8 pediatric, 1 adult and 3 mixed) were registered and received 33 lines of TTs. Median age at the beginning of TTs was 19 (range 9 to 72) and median duration between the diagnosis of osteosarcoma and the beginning of a TTs was 2.7 years (range 7 months to 7 years). A median of 3 lines of chemotherapy (range 1–8) was administrated before starting TTs (Table 1).

**Table 1:** Population characteristics

Gender			
	Male	19	66 %
	Female	10	33 %
Age at diagnosis			
		18,1	
	median (min-max)	19	8 – 65
Age at initiation of treatment			
		19	
	median (min-max)	20	9 – 72
	≤18 years	15	45 %
Histological subtype of osteosarcoma			
	osteoblastic	18	62 %
	chondroblastic	5	17 %
	osteogenic	3	10 %
	telangiectasic	2	7 %
	pleiomorphic	1	4 %
Tumor grade			
	Grade III	29	100 %
Stage at beginning of TT			
	progression	30	91 %
	complete remission after relapse	3	9 %
Localization of relapse			
	localized	0	0 %
	metastatic	24	73 %
	both	9	27 %
Number of previous treatment lines			
		2,9	
	median (min-max)	3	1 – 8
	0	0	0 %
	1	4	12 %
	2	12	36 %
	3	10	30 %
	≥4	7	21 %
Delay diagnosis - initiation of TT			
		2,8 years	
	median (min-max)	2,7 years	(0,6 – 7 years)

### Off-label targeted therapies

The decision of using off-label TTs was made in a MTB for 24 patients (73 %). There was no difference in the decision process between adults and pediatric units. Sirolimus was used for 23 patients (70 %), mostly in combination with chemotherapy (*n* = 20). Multi-targeted Tyrosine Kinase Inhibitors (TKI) were used in 10 patients (Table 2). Doses and modalities of treatment were heterogeneous.

**Table 2:** Duration of response

Targeted Therapy	*N* = 33	Stable disease as best response	Median duration of response (months)
Sirolimus alone	3	1	4,75
Sirolimus Cy	13	7 (3 maintained complete remission)	5,4
Sirolimus Cy Adriamycine	1	1	6,2
Sirolimus Cy Vinorelbine	3	0	
Sirolimus Cy Zolendronate	1	1	9
Sirolimus Irinotecan	2	0	
Sorafenib	4	3	3,1
Sunitinib	5	2 (1 PR)	3,4
Pazopanib	1	0	
Total	33	15	4,8

*Cy* cyclophosphamide

### Efficacy of targeted therapies

#### Response to treatment

Stabilization of the disease was observed in 15 patients (45.5 %, 95 % CI [28.5–62.4]), with a median duration of stabilization of 4.8 months (range 1 to 17).

Among the 20 patients in progressive disease treated with sirolimus, 7 (35 %) were stabilized: 1 with sirolimus alone, 6 in combination. Two patients treated in CR were maintained 4.8, 12.9 months respectively. The third patient stopped treatment after 17 months of continuing CR.

Under sorafenib (*n* = 4), stabilization was observed for 3 patients. One clinical PR (not RECIST) and one stabilization were observed under sunitinib. The patient treated with pazopanib had rapid disease progression (Table 2).

#### Follow up and survival

**Fig. 1:**
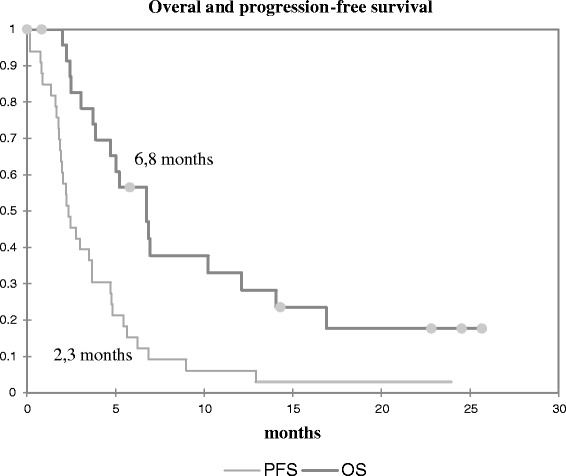
Overall survival and progression free survival

The median follow-up time after diagnosis was 3 years (range 1.1 to 7.2). The median PFS for the whole group was 2.3 months (95 % CI [1.9–3.7]). The PFS was 61 % at 2 months (*n* = 20), 30 % at four months (*n* = 10), 15 % at six months (*n* = 5) (Fig. 1).

**Fig. 2:**
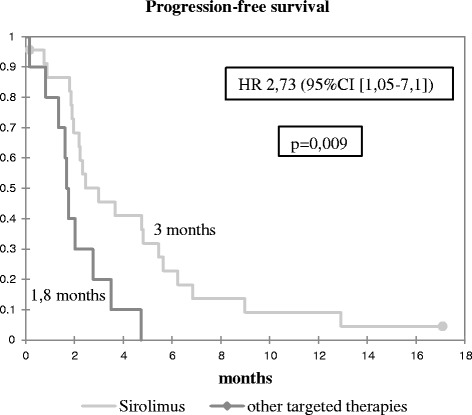
Progression-free survival according to treatment

The median PFS was 3 months (95 % CI [2.2–5.4]) for patients treated by sirolimus (2.7 months in combination, 5.7 months alone) and 1.8 months (95 % CI [1.3–2.8]) for patients receiving TKI (Fig. 2). Six-month PFS was 22 % for patients receiving sirolimus, and 0 % for other TTs. In a multivariate analysis, the only factor significantly affecting the prognosis was the TT used: patients treated by sirolimus had a better PFS, with a hazard ratio of 2.7 (95 % CI [1.05–7.1]) (Table 3).

**Table 3:** Multivariate analysis: factors influencing PFS

	Hazard Ratio	95 % CI	*p*
Delay before treatment	1,00	0,99–1	0,44
≤2 previous treatment lines	0,69	0,27 – 1,74	0,43
Histology : osteoblastic	0,80	0,37 – 1,73	0,57
Treatment by Sirolimus	2,73	1,05 – 7,1	0,04

The median PFS was 2 months (95 % CI [0.8–9]) for 4 patients treated at first relapse, 2.3 months (95 % CI [1.9–6.9]) for 12 patients experiencing a second relapse, 3 months (95 % CI [1.3–4.7]) for 10 patients at third relapse, and 2.2 months (95 % CI [1.8–3.5]) for 7 patients at fourth (or more) relapse.

Five patients achieving 6-months PFS had received the combination sirolimus-cyclophosphamide. Their median age was 17 at the beginning of TTs. One patient experienced a first relapse while the others had a second, and two were in complete remission at the treatment initiation.

The median OS was 6.8 months (95 % CI [4.7–12.1]). OS at one year was 24 % (30 % with sirolimus, 10 % with TKI).

### Tolerance of treatment

Treatment interruption occurred in 26 cases (79 %) due to disease progression and in 3 cases (9 %) due to death caused by cancer. Only one TT line was stopped for toxicities (grade 3 hematuric cystitis due to cyclophosphamide).

Among 33 lines of treatment, 22 (67 %) patients reported at least one adverse event (AE). Thirty-nine AEs were reported. Gastro-intestinal toxicity was observed in 27 % of patients (nausea, vomiting, stomatitis), hematologic toxicity in 24 % and fatigue in 24 %. Other AEs (skin, infection, headache, alopecia, depression) were reported in less than 10 % of cases (Table 4).

**Table 4:** Adverse events

	Total	Grade		
	*N*(%)	1	2	3 – 4
Sirolimus (*n* = 23)				
At least 1 toxicity reported	14 (60)			
Intestinal toxicity	8 (34)	7	1	
Skin toxicity, infections	1 (4)	1	1	
Hematologic toxicity	6 (26)	1	1	4
Urinary toxicity	1 (4)			1
Neurological toxicity	3 (13)	2	1	
Other (fatigue, pain)	6 (26)	4	2	
Dose modification	2 (9)			
Discontinuation for toxicity	1 (4)			
Sunitinib (*n* = 5)				
At least 1 toxicity reported	4 (80)			
Hematologic toxicity	2 (40)		2	
Pulmonary toxicity	1 (20)			1
Other (fatigue)	2 (40)	2		1
Dose modification	1 (20)			
Sorafenib (*n* = 4)				
At least 1 toxicity reported	3 (75)			
Skin toxicity, infections	2 (50)		1	1
other (fatigue, psychological)	4 (100)		1	2
Dose modification	2 (50)			
Pazopanib (*n* = 1)				
At least 1 toxicity reported	1			
Intestinal toxicity	1		1	
Other (fatigue)	1			1
Dose modification	0			
Total	≥1 AE: 22	17	11	11

Most of AEs were grade 1–2 (72 % of AEs). Hematologic, fatigue, and skin, grade 3, were observed for 9 patients (27 %). Grade 4 was hematologic and affected only 2 patients treated by sirolimus – cyclophosphamide – vinorelbine or adriamycine. The median grade of toxicities with TKI was 2.3, and with sirolimus 1.7.

Five TTs (15 %) were modified for toxicity (dose reduction or temporary interruption). No toxic death was reported.

## Discussion

This study reported a 45.5 % disease control rate with TTs used off-label in refractory relapsed osteosarcomas with a good tolerance profile. In a multivariate analysis, PFS seemed superior for patients receiving sirolimus compared to other TTs.

Many molecular abnormalities are identified in osteosarcomas giving the cancer cells some particular characteristics: proliferative signals (PDGFR, IGFR, c-KIT), resistance to retroaction signals (p53, RB), resistance to cell death (ERK, Bcl-2), angiogenesis (VEGFR, PDGFR), resistance to immune destruction (IFN) [12]. Potential TTs could either inhibit growth factor signaling pathways, or enhance apoptosis, or inhibit the metastatic process, or modulate the antitumor immune response, or modulate the bone microenvironment to increase local control of the primary tumor, limit metastatic spread, and finally improve patient survival [17].

mTOR is an intracellular protein, playing a major role in protein synthesis and influencing the cell growth, differentiation and apoptosis: this pathway is unregulated in many cancers, leading to the permanent activation, often under the influence of IGF1R. mTOR also plays a role in angiogenesis by controlling the production of HIF (Hypoxia Inducible Factor) [18]. Preclinical studies demonstrated that sirolimus, the main mTOR inhibitor, blocks the ezrin pathway implicated in the metastatic migration of osteosarcomas [19]. In 2012, a phase II study reported a clinical benefit in 28.8 % of patients treated with ridaforolimus for a metastatic or inoperable sarcoma with an increased PFS compared to untreated patients [20]. Another phase II study testing the association of sirolimus and cyclophosphamide in soft tissue and bone sarcomas, highlighted a synergic effect of the two drugs, leading to an increased PFS with a good tolerance [21]. A double blind phase III maintenance trial comparing ridaforolimus and placebo in advanced sarcoma after stabilization or response with chemotherapy, enrolled 50 bone sarcoma patients showing a longer PFS and a 28 % reduction in the risk of death or progression with the maintenance strategy [22]. This data constituted the rational for using mTor inhibitors in refractory osteosarcomas, first in adults and recently in pediatric population (Table 5). Data provided by OUTC’S registry confirmed the value of this agent in osteosarcomas especially combined with conventional chemotherapy to prolong survival and time to progression in this particularly dismal prognosis group.

**Table 5:** Studies reporting any benefit of TTs for osteosarcoma patients

mTOR inhibitors			
Ridaforolimus in patients with advanced bone and soft tissue sarcomas	Chawla et al.	Phase II	2012
Sirolimus and Cyclophosphamide in patients with advanced sarcomas	Schuetze et al.	Phase II	2012
Ridaforolimus versus placebo to control metastatic sarcomas in patients after benefit of prior chemotherapy (SUCCEED)	Demetri et al.	Phase III	2013
TKI			
Sorafenib blocks tumour growth, angiogenesis and metastatic potential	Pignochino et al.	preclinical	2009
Sorafenib in patients with metastatic or recurrent sarcomas	Maki et al.	Phase II	2009
Sorafenib in relapsed and unresectable high-grade osteosarcoma after failure of tandard multimodal therapy: an Italian Sarcoma Group Study	Grignani et al.	Phase II	2012
Initial testing of sunitinib by the pediatric preclinical testing program	Maris et al.	Phase I	2008
Sunitinib in pediatric patients with refractory solid tumors: a Children’s Oncology Group study	Dubois et al.	Phase I	2011
Sunitinib in patients with relapsed or refractory soft tissue sarcomas	Tariq Mahmood et al.	Phase II	2011
Pazopanib for metastatic soft-tissue sarcoma (PALETTE)	Van der Graaf et al.	Phase III	2012
Pazopanib in patients with relapsed or refractory advanced soft-tissue sarcoma	Sleijfer et al.	Phase II	2009

Sorafenib inhibits B-raf, c-KIT, PDGFR, VEGFR and RET. In osteosarcoma, sorafenib inhibits the proliferation of tumor, angiogenesis (VEGF), invasion (MMP2), the emergence of pulmonary metastases (Erzin/*β*4-integrin/ PI3K) and induces apoptosis [23]. This drug has already been approved for renal and hepatocarcinoma treatment and has shown good responses in angiosarcomas [24]. Yet, the use of sorafenib in osteosarcomas is mainly based on a phase II study, conducted in 35 patients with progression despite standard treatment and reporting 5 PRs, a clinical benefit rate of 29 % and a four-month PFS of 46 % [25].

Sunitinib inhibits FLT3, c-KIT, PDGFR and VEGF. Efficacy was observed with in vivo models, mostly pediatric tumors, including Ewing sarcoma xenografts [26]. Clinical benefit is reported for 4 patients with sarcomas in phase I [27] and 34 in phase II studies [28].

Pazopanib is mainly steered against VEGFR and PDGFR. A phase II study reported 9 cases of PR and improvement of OS and PFS for 143 patients with progressive soft tissue sarcoma [29]. A randomized double blind phase III study of pazopanib versus placebo, showed improved OS and PFS for a metastatic soft tissue sarcoma after failure of chemotherapy treatment [30]. A randomized double-blinded phase II is currently open to evaluate regorafenib, a promising TKI [31] in advanced bone sarcomas [32]. Based on this literature, TKI have been used off-label in adult refractory sarcoma first, thereafter by pediatricians influenced by adult practices despite the paucity of pharmacological data in pediatric population.

We report in this study only one objective response after initiation of TT. It has been suggested that the evaluation of TTs efficacy could not be done by RECIST compared to conventional treatments because TKI are mainly cytostatic. Some cases of cystic tumors after treatment by TKI have been reported [33]. Indeed, a stable disease induced by a TT could be considered as a satisfying response and a significant clinical benefit given the poor prognosis of metastatic refractory sarcomas. In order to guide the objectives of clinical trials, the EORTC Sarcoma Group (European Organization for Research and Treatment of Cancer) defined that a second-line treatment could be considered active if it showed a 6-month PFS of 40 % and as inactive if it was below 20 % [33]. In our study, six-month PFS was 15 % (22 % with sirolimus, 0 % with TKI), but all patients included had very poor prognosis factors: inoperable tumor, high grade histology, treatment-line failures. Most published series about this population reported dismal prognosis, with short median survival especially after several relapses [11, 34]. In this cohort, the one-year OS of 24 % and median survival of 6.8 months could be a significant result. The difference observed in median PFS between sirolimus group and TKI group (2.3 versus 1.8 months) encourages investigating this drug in a clinical trial.

Given the number of different mechanisms involved in carcinogenesis and treatment failures, a molecular study of each tumor could guide the indications of TTs and compounds. Some mechanisms lead to the cell resistance to Sirolimus, in particular because only the complex MTORC1 is sensitive to Sirolimus, whereas MTORC2 is resistant [17]. The activation of MTORC2 leads to treatment failure. This mechanism can be blocked by the association with Sorafenib: in vitro and in vivo, the combination of the two drugs increases the anti-tumor, anti-angiogenic and anti-metastatic activity [35]. Despite this data, no combination of TKI with mTOR inhibitor was reported in OUTC’S: it could be worth exploring this strategy.

In this study, tumor control lasted more than 6 months for 5 patients. These patients had a median age of 17 at the TT initiation, which is below the median age of the whole group and compatible with data showing a better response to chemotherapy in children [36]. All these patients received sirolimus in association with cyclophosphamide. One patient was treated at first relapse and the others at second relapse, suggesting that efficiency of sirolimus could be optimized when used with minimal tumoral disease.

We must underscore that three patients received a maintenance treatment combining sirolimus-cyclophosphamide, after complete remission by surgery and chemotherapy. This strategy is developing in sarcomas, supported by studies suggesting that it could improve survival and decrease the risk of relapse in high-risk patients [22, 37] and must be confirmed in randomized clinical trial dedicated to maintenance therapy including PFS, OS and quality of life.

Observed data of toxicity are similar to what was already described in clinical trials [13]. No major toxic effect has been reported and only one patient had to stop TTs because of toxicity, showing that tolerance to TTs is acceptable, even in children.

The main limitation of this study is the small number of patients, due to the rarity of these tumors, which can reduce the statistical power, in particular for the comparison between TKI and sirolimus (since the CI of the hazard ratio approximates 1). The specificities of pediatric population make it difficult to launch clinical trials assessing efficacy of TTs in osteosarcomas. Registering patient in a national database like OUTC’S is an opportunity to obtain more information about safety and efficacy of drugs used off-label with a rational based on published data.

## Conclusion

Targeted therapies could play a part in the treatment of refractory osteosarcomas or in maintenance for patients with a high risk of relapse. Tolerance is acceptable, even for patients under 18. This data suggests that sirolimus could have an interesting anti-tumor activity in osteosarcomas and deserves to be evaluated in a prospective trial, either alone or in combination with chemotherapy.

## Abbreviations

AE: adverse events
CDK: Cyclin dependent kinase
c-KIT: v-kit Hardy-Zuckerman 4 feline sarcoma viral oncogene homolog
CNIL: Commission national de l’informatique et des libertés
CR: complete response
EORTC: European Organization for Research and Treatment of Cancer
ERK: extracellular signal-regulated kinase
FLT3: Fms-like tyrosine kinase 3
GADD45: Growth arrest and DNA damage-inductible 45 protein
GSF-GETO: French sarcoma group, Group for the study of bone tumors
HIF1: Hypoxia inductible factor
IC: confidence interval
IFN: Interferon
IGFR: Insulin-like growth factor receptor
MMP2: matrix metallopeptidase 2
MTB: Multidisciplinary tumor board
mTOR: mammalian target of rapamycin
MTORC: mammalian target of rapamycin complex
NetSarc: French Sarcoma Network
OS: overall survival
OUTC’S: National observatory for the off-label use of targeted therapies in sarcomas
PDGFR: Platelet-derived growth factor receptor
PFS: progression-free survival
PI3K: phosphoinositide-3-kinase
PR: partial response
RANK/RANKL/OPG: Receptor activator of NF-KappaB / Receptor activator of NF-KappaB ligand / osteoprotegerin protein
RB: Retinoblastoma protein
RECIST: Response Evaluation Criteria for Solid Tumors
RET: rearranged during transfection proto-oncogene
SD: stable disease
SHH/GLI: Sonic Hedgehog
TKI: tyrosine kinase inhibitors
TT: targeted therapy
VEGFR: Vascular endothelial growth factor receptor
